# Effect of Fermented* Spirulina maxima* Extract on Cognitive-Enhancing Activities in Mice with Scopolamine-Induced Dementia

**DOI:** 10.1155/2018/7218504

**Published:** 2018-11-26

**Authors:** Woon Yong Choi, Do Hyung Kang, Hyeon Yong Lee

**Affiliations:** ^1^Department of Biomedical Materials Engineering, Kangwon National University, Chuncheon 200-701, Republic of Korea; ^2^The Jeju International Marine Science Center for Research & Education, Korea Institute of Ocean Science and Technology (KIOST), Ansan, Republic of Korea; ^3^Department of Food Science and Engineering, Seowon University, Cheongju, 361-742, Republic of Korea

## Abstract

This work provides the first demonstration that* Spirulina maxima* extract fermented with the lactic acid bacterium* Lactobacillus planetarium* HY-08 has the ability to ameliorate scopolamine-induced memory impairment in mice. The fermented extract exhibited good cognitive-enhancing activities, as demonstrated through Morris water maze and passive avoidance experiments: in these tests, the mice administered the fermented extract at a dose of 400 mg/kg exhibited an escape latency time and a latency time of 88.5 and 76.0 sec, respectively, whereas those administered donepezil, which was used as a positive control, showed an escape latency time and a latency time of 81.3 and 83.3 sec, respectively. However, an extract of 200 mg/kg was considered economically feasible for maintaining relatively high memory-improving activities because only a slight difference in activities was found between 200 and 400 mg/kg. The study also provides the first demonstration that *β*-carotene, one of the major bioactive substances in* S. maxima*, has memory-enhancing activity. A detailed analysis of the mechanism for the cognitive-enhancing activities of the fermented extract revealed that the fermented extract effectively increased the phosphorylation of both extracellular signal-regulated kinases (p-ERK) and p-cAMP response element-binding protein (p-CREB) and sequentially upregulated the expression of brain-derived neurotrophic factor (BDNF), whose signaling pathway responds to a reduction in oxidative stress in the brain. The results indicate that the improved efficacy of the fermented extract was likely due to the synergistic effects of *β*-carotene and other bioactive substances. Therefore, it can be concluded that the fermented extract exerts memory-improving effects in the hippocampus of scopolamine-treated mice through an initial increase in ERK signaling and a sequential induction of the expression of p-CREB and BDNF, and these effects are related to the antioxidant activities of *β*-carotene and other components.

## 1. Introduction

Many recent technological developments have extended the human lifespan, and as a result, dementia, a degenerative brain disease, has received increased attention [1]. Oxidative stress, which is one of the causes of dementia and hypomnesis, is caused by an imbalance between the production of reactive oxygen species (ROS) and the antioxidative capacity, which leads to the occurrence of many elements, such as amyloid-*β*, cytokines, excitotoxic amino acids, nitric oxide, and abnormalities in the mitochondrial electron transport system [2, 3]. Therefore, oxidative stress is associated with diseases such as ischemia and degenerative brain diseases (Alzheimer's disease, Parkinson's disease, and amyotrophic lateral sclerosis) [2, 3]. Studies on diverse antioxidant substances have aimed to improve the conditions of oxidative stress-induced dementia and hypomnesis caused by ROS [3, 4]. Prolyl endopeptidase (PEP), donepezil, rivastigmine, and galantamine have been developed as therapeutic agents for degenerative brain diseases and have been sold for improving the memory of demented patients depending on their mechanisms of action. However, these drugs have side effects, such as hepatotoxicity, vomiting, diarrhea, and gastrointestinal disorders [5–7]. Therefore, researchers have actively attempted to develop natural product-derived functional foods without side effects [8, 9].

*S. maxima* is known to contain many physiologically active substances, such as chlorophylls, carotenoids, various sulfated polysaccharides, and vitamin B12, as well as C-phycocyanin (C-PC), which is a representative active substance [10, 11]. In particular, *β*-carotene, which is one of the many bioactive substances in* Spirulina*, has been reported to exert memory-improving effects on Alzheimer-type hypomnesis models due on its antioxidant activity [12, 13]. However, the cognitive-enhancing activities of the whole extract of* Spirulina* in comparison to those of *β*-carotene have not been extensively investigated. In addition, recent studies have intensively investigated the various beneficial effects of the microbiome, particularly lactic acid bacteria [14]. The findings of these studies include the memory-improvement effects of lactic acid bacteria and its fermented products, and although these effects are not very high, the high efficacy of these substances should be considered very important for various medical applications due to the difficulties of managing the associated chronic diseases [15, 16]. Therefore, by assessing the up- and downregulation of proteins involved in memory improvements observed in animal experiments, this study aimed to understand the mechanisms of the cognitive-enhancing activities of* S. maxima* extract fermented with a lactic acid bacterium.

## 2. Materials and Methods

### 2.1. Preparation of Fermented S. maxima Extracts

Samples of* Spirulina maxima *cultivated with volcano sea water in Jeju, Korea, were supplied by the Korea Institute of Ocean Science & Technology (KIOST, Jeju, Korea). First, 100 g of dried* S. maxima* powder was mixed with 1 L of distilled water, extracted at 80°C for 6 h, and centrifuged at 8000 rpm for 20 min to remove the solids. Culture medium consisting of 0.5 (%) yeast extract, 1% peptone, 2% glucose, 0.01% magnesium sulfate, 0.005% manganese sulfate, 0.2% potassium phosphate, and 0.1% (w/v) polysorbate 80 was then added to the supernatants. The medium was subsequently inoculated with* Lactobacillus plantarum* HY-08 (registered in the National Center for Biotechnology Information (NCBI) GenBank, USA, as MG547899) with an initial seed volume of 1 x 10^5^ CFU/mL and further incubated under anaerobic conditions at 37°C until the final cell counts reached 1 x 10^8^ CFU/mL. The fermentation was then stopped, and the culture broth was sterilized at 121°C for 15 min. After sterilization, the whole broth was processed with a spray drier (Spray Dryer B-290, Buchi AG, Flawil, Switzerland) with a feeding temperature of 180°C until the culture broth completely dried. The dried powder was then stored at 4°C prior to the experiments.

### 2.2. Experimental Animals

Four-week-old ICR male mice weighing approximately 25-30 g (Orient Co., Seongnam, Korea) were first placed in a breeding room under the following conditions: temperature of 22 ± 2°C, 50 ± 5% humidity, and 12-h dark/12-h light cycle. The mice were adapted to the environment for one week with water and feed supplied ad libitum. The animals were then divided into four groups with six animals per group: the control group, which was administered saline; the scopolamine group, which was administered scopolamine ((−)-scopolamine hydrobromide trihydrate, S1875, Sigma-Aldrich, St. Louis, MO, USA) intraperitoneally at a dose of 1 mg/kg to induce memory impairment; the donepezil group, which was administered 6 mg/kg donepezil (D6821, Sigma-Aldrich, St. Louis, MO, USA); and the positive control group, which was administered 1 mg/kg and 5 mg/kg *β*-carotene (C4582, Sigma-Aldrich, St. Louis, MO, USA) [17, 18]. The fermented* S. maxima* extract was also administered at doses of 50, 100, 200, and 400 mg/kg with saline water.

### 2.3. Morris Water Maze Test

First, spatial memory was assessed using the Morris water maze experiment as follows [17, 18]: A water maze pool with a diameter of 90 cm and a height of 40 cm was filled with water (20 ± 1°C), and milk was mixed into the water until the solution became sufficiently opaque that the platform was invisible. The pool was then divided into four sections, and the platform (10-cm diameter and 26-cm height) was set up in the middle section at 1 cm below the water surface. The experiments were performed for 4 days. To ensure that the point of entrance into the water was changed daily, the section of the experiment was changed each day from east to west and south to north. The mice were allowed to swim for 60 sec without a platform on the first day of the experiment to allow the mice to adapt to the pool, and measurements were conducted starting on the second day. During an experiment, a mouse was allowed to stay on the platform for 10 sec after arriving, and if a mouse was unable to find the platform for 120 sec, it was placed on the platform for 10 sec to help the mouse remember the location of the platform before the next experiment was conducted. The time from the moment of a mouse's entrance into the water to its arrival at the platform was measured as the escape latency time [17, 18].

### 2.4. Passive Avoidance Test

Passive avoidance experiments were conducted using passive avoidance boxes (GEMINITM Avoidance System, San Diego Instruments, San Diego, CA, USA) as follows [17, 18]: A door through which mice can move was installed between two boxes composed of acryl, and a stainless steel rod was installed on the floor to allow generation of an electric charge. In the first experiment, the mice were placed in one box and allowed to adapt for 1 min to a dark environment. The light in the box was then turned on, and noise was generated simultaneously to encourage the mouse to move to the opposite acrylic box. The mouse was then placed in the dark acrylic box for 20 sec before the light was turned on and noises were generated to encourage the mouse to move into the opposite avoidance box, and 2 sec later, the mouse was administered an electric shock (0.1 mA/10 g body). Twenty-four hours later, the same experiment was conducted, and the time from the start of the experiment to the time at which the mouse moved to the avoidance box was measured. The experiment was terminated if the mouse did not move from the first box for 180 sec [17, 18].

### 2.5. Measurement of Acetylcholine Esterase Activities in Hippocampal Tissues of Scopolamine-Treated Mice

Acetylcholine esterase (AChE) activities in the tissue were also measured as follows [18, 19]: After the animal behavior experiments, the hippocampus of the mice was incised from the brain and rapidly homogenized with solutions composed of 33 *μ*L of homogenate, 470 *μ*L of sodium phosphate buffer, and 167 *μ*L of 5,5′-dithiobis(2-nitrobenzoic acid) (DTNB) (D8130, Sigma-Aldrich, St. Louis, MO, USA). Then, 280 *μ*L of acetylcholine iodide was added to the reaction mixture. After incubation, the absorbance of the reaction at 412 nm using a spectrophotometer and the activities of AChE were estimated as the optical density value per mg of protein [18, 19].

### 2.6. Western Blot Analysis of BDNF, CREB, and ERK Expression

Immediately after the animal behavioral experiments, all of the experimental animals were sacrificed, and the hippocampal tissues were extracted. The proteins were then separated and quantified with a Bradford assay. Supernatant containing 50 *μ*g of protein was subjected to 12% SDS-polyacrylamide gel electrophoresis (SDS-PAGE) at 100 V for 3 h, and the proteins were then transferred to a polyvinylidene fluoride (PVDF, Bio-Rad, Hercules, CA, USA) membrane. The membrane was blocked with 5% skim milk for 1 h and incubated with a 1:2000 dilution of *β*-actin antibody (SC-1616, Santa Cruz Biotechnology, TX, USA), a 1:1000 dilution of cAMP response element-binding protein (CREB-1) antibody (SC-240, Santa Cruz Biotechnology, TX, USA), a 1:500 dilution of p-CREB antibody (SC-81486, Santa Cruz Biotechnology, TX, USA), a 1:1000 dilution of brain-derived neurotrophic factor (BDNF) antibody (SC-546, Santa Cruz Biotechnology, TX, USA), and a 1:1000 dilution of extracellular signal-regulated kinase (ERK1/2) antibody (SC-135900, Santa Cruz Biotechnology, TX, USA) at 4°C for 24 h. The membrane was subsequently washed three times with 0.1% phosphate buffered saline with Tween 20 (PBST) (P3813, Sigma-Aldrich, St. Louis, MO, USA), incubated with the secondary antibodies goat-anti-rabbit IgG HRP (SC-2004, Santa Cruz Biotechnology, TX, USA), which was used at 1:2000 dilution identify BDNF antibody, donkey-anti-goat IgG HRP (SC-2020, Santa Cruz Biotechnology, TX, USA), which was used at 1:2000 dilution to identify *β*-actin antibody, and goat-anti-mouse IgG HRP, which was used at 1:2000 dilution to identity CREB-1, p-CREB, and ERK1/2, for 2 h at room temperature and washed three times with 0.1% PBST. The plate was then sprayed with an enhanced chemiluminescence (ECL) buffer for 1 min in several directions and visualized using a ChemiDoc™ Imaging System (Bio-Rad, CA, USA) [18, 20].

### 2.7. Statistical Analysis

The data are expressed as the means ± SDs (standard deviations), and the means were calculated as the averages from five results per experiment. The data were also analyzed by one-way analysis of variance (ANOVA), and the differences in the mean values were considered significantly different if* p*<0.05,* p*<0.01 and* p*<0.001.

## 3. Results and Discussion

### 3.1. Cognitive-Enhancing Activities of the Fermented S. maxima Extract

**Figure 1:**
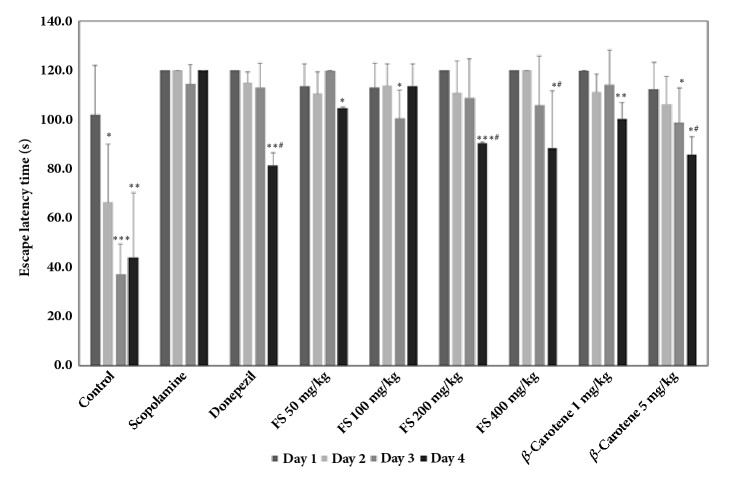
Escape latency time of the following groups of mice in the Morris water maze test: control group, scopolamine-treated mice, scopolamine-treated mice administered donepezil, scopolamine-treated mice administered 50, 100, 200, and 400 mg/kg fermented* Spirulina maxima *extract (FS), and scopolamine-treated mice administered 1 and 5 mg/kg *β*-carotene. The values shown are the mean escape latency times ± SDs (n=6). *∗p*<0.05, *∗∗p*<0.01, and *∗∗∗p*<0.001 show significant differences in comparison to the scopolamine-treated group (n=6). ^#^*p*<0.05, ^##^*p*<0.01, and ^###^*p*<0.001 show significant differences compared with the control group (n=6).

**Figure 2:**
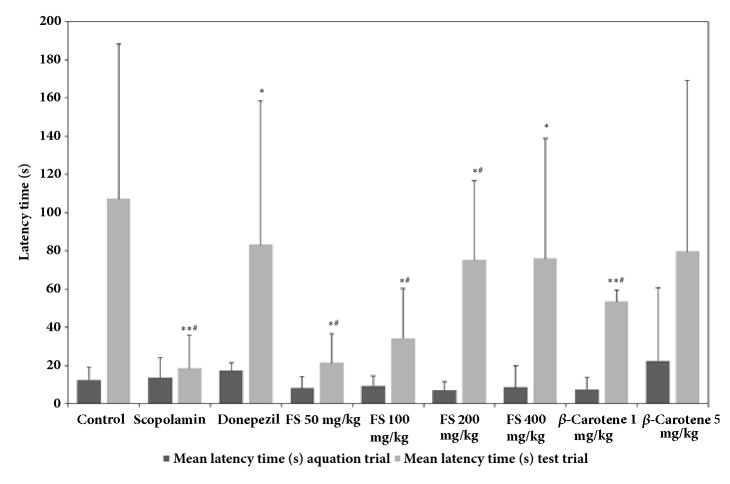
Comparison of the latency times of the following groups of mice in the passive avoidance test: control group, scopolamine-treated mice, scopolamine-treated mice administered donepezil, scopolamine-treated mice administered 50, 100, 200, and 400 mg/kg fermented* Spirulina maxima *extract (FS), and scopolamine-treated mice administered 1 and 5 mg/kg *β*-carotene. The values shown are the mean escape latency times ± SDs (n=6). *∗p*<0.05, *∗∗p*<0.01, and *∗∗∗p*<0.001 show significant differences in comparison to the scopolamine-treated group (n=6). ^#^*p*<0.05, ^##^*p*<0.01, and ^###^*p*<0.001 show significant differences compared with the control group (n=6).

As shown in Figure 1, the spatial memory-improving effect of the fermented* S. maxima* extract was evaluated by measuring the escape latency time through Morris water maze experiments. The results showed that the mean escape latency of the control group decreased over the 4-day experimental period, whereas the mice in the scopolamine-induced group failed to find the platform or require a notably longer time to find the platform on all four days. However, the escape latency time of all the groups administered the fermented* S. maxima* extract started to decrease after the third experimental day and was greatly reduced on the fourth day. The results also showed that the escape latency time was decreased by 88.5 sec through the administration of the extract at 400 mg/kg, and decreases of 104.7, 110.8, and 91.5 sec were obtained after the administration of the extract at 50, 100, and 200 mg/kg, respectively. Notably, the effect of 400 mg/kg extract (escape latency time of 88.5 sec) was not much lower than that of donepezil (positive control, 81.3 sec), which could imply that the fermented extract definitely improved the spatial memory deficit. Moreover, the administration of donepezil greatly decreased the latency time on the fourth experimental day, which indicated that the effect of donepezil started to be observed after three days of administration. A similar tendency was observed in all the groups treated with the extracts, which could also indicate that the effect of the extract is somewhat similar to that of donepezil. In addition to the effects of the fermented extract, the efficacy of *β*-carotene, which is one of most abundant bioactive substances in* Spirulina*, was also observed in the groups treated with 1 and 5 mg/kg pure *β*-carotene, which was used as a positive control. These dosages were selected based on the following: most species of* Spirulina* [21, 22] contain approximately 100 mg/kg *β*-carotene, and based on an extraction yield of 20% (w/w) (data not shown), 200 mg of fermented extract was calculated to contain 1 mg of pure *β*-carotene. Interestingly, as determined through the water maze test, the effect of 200 mg/kg fermented extract (90.5 sec) was better than that 1 mg/kg *β*-carotene (100.2 sec), although both values were within the confidence intervals of the other group, and was also similar to that of 5 mg/kg *β*-carotene (85.8 sec). This result could strongly imply a synergistic effect between *β*-carotene and other bioactive substances in the fermented extract because the effect of 200 mg/kg extract was better than that of 1 mg/kg *β*-carotene, which corresponds to the amount of pure *β*-carotene in the same dose of the extract, and similar to the that of 5 mg/kg *β*-carotene. Therefore, these results indicate that the fermented extract has potential memory-improving ability. However, the effect of the extract at a dose of 400 mg/kg was, interestingly, not notably higher than that of the extract at 200 mg/kg, whereas treatment with 50 and 100 mg/kg yielded dose-dependent decreases in the escape latency time on the fourth day. This finding implies that a dose of 200 mg/kg might be a more economically feasible dose of the fermented extract that yields the maximal effects. As shown in Figure 2, passive avoidance tests were also conducted to assess the long-term memory-improving effects of the fermented extract. The control group (untreated group) showed a latency time of 107.2 sec, and the mice with scopolamine-induced dementia showed a latency time of 18.4 sec. The groups treated with the fermented extracts at doses of 50 mg/kg to 400 mg/kg showed notable dose-dependent increases in the latency time ranging from 21.5 to 76.0 sec. Specifically, a substantial increase in the mean latency time was observed after the administration of 200 mg/kg extract (75.3 sec); however, the administration of 400 mg/kg extract, which was the highest dosage used in this study, resulted in a latency time of 76.0 sec. This response was similar to that obtained with the water maze test, as shown in Figure 1: less improvement was obtained with 400 mg/kg. In general, the experimental errors associated with the estimations of the latency times of each group appeared to be high, which might have been due to the characteristics of the passive avoidance test [23]. However, the differences in latency time among the groups were higher than the errors within the group, which indicates that the extracts definitely exerted cognitive-enhancing effects on scopolamine-treated mice. Therefore, the results from both tests could indicate the existence of an optimal or more effective dosage of the fermented extract with respect to improvements in memory deficiency. As positive controls, 6 mg/kg donepezil showed the best efficacy, with a latency time of 83.3 sec, whereas 1 and 5 mg/kg *β*-carotene yielded latency times of 53.6 and 79.8 sec, respectively. However, the mean latency time of 53.6 sec obtained with 1 mg/kg *β*-carotene was notably shorter than that of 75.3 sec obtained with 200 mg/kg fermented extract, even though this dosage of *β*-carotene was assumed to be equal to the amount of pure *β*-carotene administered with 200 mg/kg fermented extract. In addition, 5 mg/kg *β*-carotene, which was the highest dosage tested, resulted in a mean latency time of 79.8 sec, which was somewhat lower than the mean latency time of 83.3 sec obtained with donepezil, but *β*-carotene clearly has a memory-enhancing effect, similarly to the results obtained in previous studies. More specifically, combination treatment consisting of 5.6 to 16.7 mg/day of *β*-carotene with vitamin C or E improve the cognitive abilities of elderly men [13], which shows that *β*-carotene could help manage Alzheimer's disease in elderly patients due to its strong antioxidant activities. However, in most studies on the effects of *β*-carotene, enhancements in cognitive activities were not observed with after treatment with only *β*-carotene but rather after treatment with *β*-carotene in combination with other supplements, such as vitamins [13, 24]. Therefore, the results of this study provide the first demonstration of the cognitive-enhancing effect of *β*-carotene administered as a single treatment. Moreover, the results shown in Figure 2 exhibited good consistency with the data presented in Figure 1, which could support the hypothesis that the extract exerts a synergistic effect due to the various bioactive components in the extracts, as demonstrated by the significant differences among the experimental groups. These results provide useful information on the merits of using crude extracts for the development of functional foods rather than single components that are highly or complexly purified from extracts through expensive and nonefficient purification steps. Interestingly, the fermented extract generally showed better efficacy in the passive avoidance experiment than in the water maze test, which implies that the extract exerted a better effect on improving long-term rather than spatial memory. This result also supports the hypothesis that the fermented extract could play a more important role in enhancing memory deficits and dementia. Moreover, because* S. maxima* contains *β*-carotene, a well-known antioxidant, the fermented* S. maxima* extract might exert memory-improving effects due to the strong antioxidant effect of *β*-carotene, as shown in Figures 1 and 2 [12, 13].

### 3.2. Inhibition of Acetylcholinesterase Activity by the Fermented Extract

As shown in Table 1, the effects of the fermented extract might involve the inhibition of acetylcholine esterase (AChE) activity because AChE has been strongly associated with memory impairment through the degradation of acetylcholine, which is a neurotransmitter in the cholinergic system of the brain [25]. First, AChE activity in the hippocampus was significantly increased in scopolamine-treated mice compared with the untreated control group. However, donepezil definitely increased the memory impairment, as demonstrated by its greater effects compared with those found in the extract-administered and *β*-carotene-treated groups. The fermented extract also inhibited AChE activities in a dosage-dependent manner, but the dose of 400 mg/kg did not exhibit a notably higher effect compared with hat of 200 mg/kg, which is consistent with the results shown in Figures 1 and 2. These findings indicate the existence of saturation and/or a most effective concentration of the fermented extract with respect to memory-enhancing activities, and thus, there might be an economically feasible dosage of the crude extracts that would not yield a reduced efficacy in industrial fields. Considering the effect of *β*-carotene on the degradation of AChE activity, the efficacy of 5 mg/kg *β*-carotene was similar to that of the extracts at 200 mg/kg or 400 mg/kg, which contain lower doses of *β*-carotene than that administered with 5 mg/kg *β*-carotene. Therefore, the fermented extract reversed the cognitive impairments through a mechanism involving the muscarinic cholinergic receptor. This result also provided strong evidence showing that the crude extract exerted better cognitive-enhancing effects than treatment with the same concentration of pure *β*-carotene, potentially due to its synergistic effects.

### 3.3. Effects of the Extracts on Upregulating BDNF, p-CREB, and p-ERK Expression

**Figure 3:**
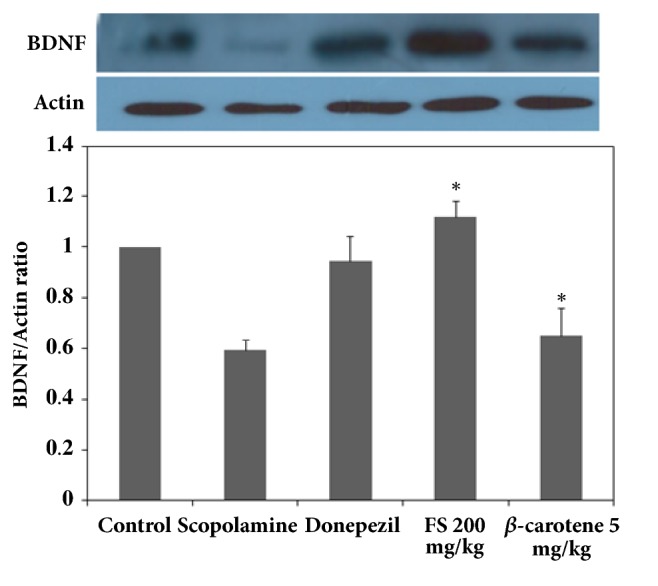
Comparison of the expression levels of brain-derived neuronal factor (BDNF) in the following groups of mice: control group, scopolamine-treated mice, scopolamine-treated mice administered donepezil, scopolamine-treated mice administered 50, 100, 200, and 400 mg/kg fermented* Spirulina maxima *extract (FS), and scopolamine-treated mice administered 1 and 5 mg/kg *β*-carotene. The values shown are the mean escape latency times ± SDs (n=6). *∗p*<0.05, *∗∗p*<0.01, and *∗∗∗p*<0.001 show significant differences in comparison to the scopolamine-treated group (n=6).

**Figure 4:**
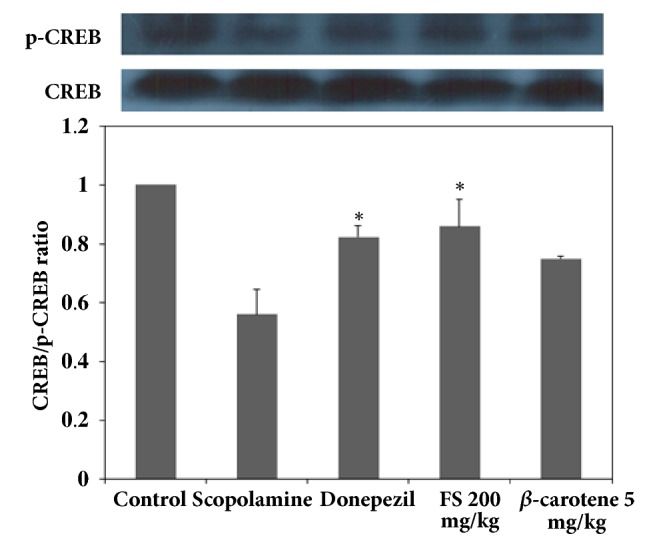
Comparison of the ratio of cAMP response element-binding (CREB) protein to phosphorylated CREB (p-CREB) protein in the following groups of mice: control group, scopolamine-treated mice, scopolamine-treated mice administered donepezil, scopolamine-treated mice administered 50, 100, 200, and 400 mg/kg fermented* Spirulina maxima *extract (FS), and scopolamine-treated mice administered 1 and 5 mg/kg *β*-carotene. The values shown are the mean escape latency times ± SDs (n=6). *∗p*<0.05, *∗∗p*<0.01, and *∗∗∗p*<0.001 show significant differences in comparison to the scopolamine-treated group (n=6).

**Figure 5:**
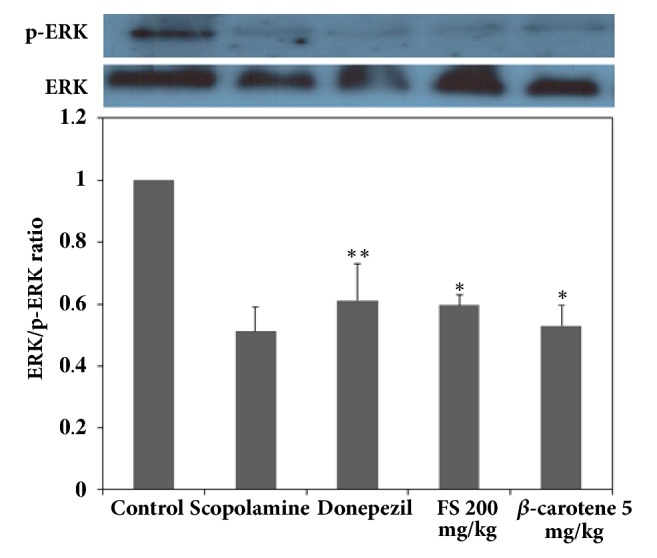
Comparison of the ratio of extracellular signal-regulated kinases (ERK) to phosphorylated ERK (p-ERK) in the following groups of mice: control group, scopolamine-treated mice, scopolamine-treated mice administered donepezil, scopolamine-treated mice administered 50, 100, 200, and 400 mg/kg fermented* Spirulina maxima *extract (FS), and scopolamine-treated mice administered 1 and 5 mg/kg *β*-carotene. The values shown are the mean escape latency times ± SDs (n=6). *∗p*<0.05, *∗∗p*<0.01, and *∗∗∗p*<0.001 show significant differences in comparison to the scopolamine-treated group (n=6).

The suppression of BDNF expression and p-CREB phosphorylation due to internal and external reasons should greatly affect long-term memory impairment [26, 27]. The activation of BDNF and CREB expression is essential for recovering and maintaining memory-improving activities [26, 27]. It is also known that various antioxidants could play important roles in the activation of these neurotransmission factors by upregulating ERK signaling and phosphorylation because ERK signaling stimulates the cascading activation of CREB phosphorylation and BDNF expression as well as protein kinase A- (PKA-) dependent long-term potentiation (LTP) [20, 27, 28]. Therefore, the mechanism underlying the memory-improving activities of the fermented extract could be associated with the activation of CREB phosphorylation through the phosphorylation of ERK, and this effect eventually upregulates BDNF expression, as demonstrated in the above-mentioned results, which showed that the fermented extract had cognitive-enhancing activities [26, 27]. First, as shown in Figure 3, the expression of BDNF in the groups administered scopolamine and donepezil as a positive control was compared. The results showed that the extract at a dose of 200 mg/kg achieved the best results, as shown in Figures 1 and 2, and 5 mg/kg *β*-carotene yielded better results than 1 mg/kg *β*-carotene. As shown in Figure 3, the lowest expression of BDNF was detected in the scopolamine-treated group, and a higher BDNF expression was observed in the group administered 200 mg/kg extract than in the untreated control group. The expression of BDNF was not markedly higher or lower in the group treated with donepezil compared with the group administered 200 mg/kg fermented extract. This result was somewhat different from the findings shown in Figures 1 and 2, which indicates that the performance of the mice administered donepezil was better than that of the groups treated with the various dosages of the fermented extract. It can be speculated that the extract more directly activated the expression of BDNF in the hippocampus than donepezil, but the in vivo response to the extract was slightly slower than that to donepezil. Interestingly, the amount of BDNF expressed in the hippocampus of the mice was not notably higher than that of the 200 mg/kg extract-treated and donepezil-treated mice. In addition, *β*-carotene must efficiently upregulate BDNF expression, as demonstrated by the notably higher expression of BDNF observed in the *β*-carotene-treated mice than in the scopolamine-treated mice. Therefore, these results definitely confirmed that the fermented extract increases the expression of BDNF at doses above 200 mg/kg, and these increases in BDNF expression resulted in better performances in both the water maze and the passive avoidance experiments. To confirm that the mechanism of action of the extracts involves the upregulation of BDNF expression in the hippocampus of mice, the activation of CREB and ERK phosphorylation was also assessed, as shown in Figures 4 and 5, because the phosphorylation of ERK and CREB should play more important roles in neuronal transmission and because the phosphorylation of ERK sequentially induces the phosphorylation of CREB, which should result in an increase in BDNF expression in the hippocampus [18, 20]. As clearly shown in Figure 5, the fermented extract has the ability to increase ERK signaling by increasing p-ERK expression in the scopolamine-treated mice, and this effect of the extract was higher than those of the positive controls donepezil and *β*-carotene, as demonstrated by the similar patterns observed in Figure 3. The activation of p-ERK also upregulated the expression of p-CREB, as shown in Figure 4. The highest expression of p-CREB was obtained with the administration of 200 mg/kg extract, which showed a similar activity to donepezil, and this result is the opposite of the results shown in Figure 4. This finding implies that the response of p-CREB expression to the extract might be less sensitive than that of p-ERK because p-CREB and BDNF participate in several signaling pathways. However, it is obvious that the fermented extract can stimulate ERK signaling, which results in the phosphorylation of CREB and thereby the induction of its transcriptional activity. In mice with scopolamine-induced memory deficiency, this response resulted in an increased expression of BDNF and eventually an improved performance. Interestingly, the effects of 5 mg/kg *β*-carotene were not better than those of 200 mg/kg extract, which contains approximately 1 mg/kg pure *β*-carotene, even though the cognitive-enhancing activities of *β*-carotene were well confirmed in the above results and are likely due to its strong antioxidant activities. Therefore, these results show that the fermented* S. maxima *extract can effectively upregulate the expression of both p-CREB and BDNF by activating p-ERK signaling in the hippocampus of scopolamine-treated mice, which eventually results in an enhancement in cognitive activities.

## 4. Conclusion

The cognitive-enhancing activities of* Spirulina maxima* extract fermented with a lactic acid bacterium were first confirmed through both Morris water maze and passive avoidance experiments. Specifically, the performance of scopolamine-treated mice was better in the passive avoidance experiments than in the water maze test, which implies that the fermented extract can more effectively improve long-term memory deficits. A fermented extract dosage of 200 mg/kg can be considered an economically feasible dosage because no significant improvements were observed with the dosage of 400 mg/kg. The efficacy of 1 mg/kg pure *β*-carotene, which corresponds too he amount of this compound in 200 mg/kg extract, was lower than that of 200 mg/kg fermented extract, and 200 mg/kg fermented extract showed similar effects to 5 mg/kg pure *β*-carotene. This result strongly indicates that the higher efficacy of the extract might be attenuated by the synergistic effects of *β*-carotene and other biologically active substances, and this finding supports the selection of crude extracts rather than the pure single compounds required by most processes, which would also impact the costs of functional foods. A detailed mechanism of the memory-improving effects of the fermented extract was then found to be strongly correlated with the activation of the p-ERK/p-CREB/BDNF pathway and the inhibition of AChE activity, and these effects should efficiently reduce internal or external oxidative stress, which is the main cause of memory impairments. Therefore, the results of this study indicate that the fermented* S. maxima* extract exerts memory-improving effects, and these effects are potentially related to the antioxidant activities of *β*-carotene and other active substances in the extracts. However, detailed studies should be performed to identify a more accurate synergistic mechanism for improving memory deficits that involves the combination of several key compounds in the fermented extract.

## Figures and Tables

**Table 1:** Effect of the fermented extracts on the inhibition of acetylcholine esterase (AChE) activities in the hippocampus of mice with scopolamine-induced memory impairment.

Groups	AChE activity (U/mg protein)
Control	0.65±0.012
Scopolamine	0.91±0.022^#^
Donepezil	0.61±0.039^*∗*^
Fermented *S. maxima *extract (50 mg/kg)	0.83±0.018^#^
Fermented *S. maxima *extract (100 mg/kg)	0.77±0.020^*∗*,#^
Fermented *S. maxima *extract (200 mg/kg)	0.69±0.051^*∗∗*^
Fermented *S. maxima *extracts (400 mg/kg)	0.68±0.045^*∗∗*^
*β*-carotene (1 mg/kg)	0.75±0.019^#^
*β*-carotene (5 mg/kg)	0.70±0.033^*∗∗*^

The values shown are the mean escape latency time ± SD (n=6). *∗p*<0.05, *∗∗p*<0.01, and *∗∗∗p*<0.001 significant in comparison to scopolamine group (n=6). ^#^*p*<0.05, ^##^*p*<0.01, and ^###^*p*<0.001 significant in comparison to control group (n=6).

## Data Availability

The data used to support the findings of this study are available from the corresponding author upon request.
